# Carcinoma arising within bilateral benign fibroepithelial tumors of the breast: a case report

**DOI:** 10.3389/fonc.2026.1966791

**Published:** 2026-09-16

**Authors:** Siheng Liu, Jie Han, Yufei Wang

**Affiliations:** 1 School of Clinical Medicine, Jining Medical University, Jining, Shandong, China; 2 Department of Breast Surgery, The First People’s Hospital of Jining, Jining, Shandong, China

**Keywords:** benign phyllodes tumor, breast fibroepithelial tumor, ductal carcinoma in situ, fibroadenoma, intratumoral malignant transformation, invasive ductal carcinoma

## Abstract

**Background:**

Malignant transformation within breast fibroepithelial tumors is uncommon, and carcinoma arising within bilateral benign fibroepithelial tumors is exceptionally rare. Preoperative imaging and needle biopsy may fail to identify small malignant foci. This report aims to expand the clinical experience regarding the diagnosis and management of this rare presentation.

**Case presentation:**

A 53-year-old perimenopausal woman presented with a large, painless mass in the right breast and had no family history of breast cancer. Mammography and ultrasonography demonstrated a large confluent mass in the right breast and multiple small nodules in the left breast, without typical malignant calcifications. The patient declined core needle biopsy and underwent wide local excision of the right breast mass and ultrasound-guided vacuum-assisted excision of a left breast lesion. Permanent histopathologic examination with immunohistochemistry revealed a benign phyllodes tumor containing a focal grade 1 invasive carcinoma of no special type in the right breast and adenosis with fibroadenoma containing focal low-grade ductal carcinoma *in situ* (DCIS)in the left breast. Both malignant foci were very small, strongly hormone receptor positive, and had low proliferative activity. Following multidisciplinary discussion, sentinel lymph node biopsy was omitted, and long-term endocrine therapy was initiated with toremifene during the perimenopausal period, with planned transition to letrozole after menopause. No recurrence was detected during 6 months of postoperative follow-up.

**Conclusion:**

Occult carcinoma can arise within bilateral benign breast fibroepithelial tumors in middle-aged and older women. Imaging may lack typical malignant features, and needle biopsy may miss small malignant foci; definitive diagnosis may therefore depend on complete excision and histopathologic evaluation. Treatment should be based on surgical excision and individualized systemic therapy according to the pathologic stage and molecular phenotype, with long-term surveillance.

## Introduction

Global cancer statistics indicate that approximately 2.3 million new cases of female breast cancer and 670, 000 related deaths occurred worldwide in 2022. Breast cancer has surpassed lung cancer as the most commonly diagnosed malignancy among women globally (1). In China, the incidence of breast cancer has also continued to rise, with a trend toward diagnosis at younger ages, posing a substantial threat to women’s health and quality of life.

Breast fibroepithelial tumors are biphasic neoplasms composed of epithelial and stromal components and include the common fibroadenoma and the less common phyllodes tumor (2). Fibroadenomas are hormonally responsive lesions composed of mixed epithelial and stromal elements. Phyllodes tumors(PTs) are well-circumscribed biphasic fibroepithelial neoplasms characterized by epithelial-lined clefts surrounded by a typically hypercellular, leaf-like stroma and have a recognized potential for local recurrence. Their biologic behavior differs fundamentally from that of epithelial breast carcinomas, and no direct causal oncogenic relationship has been established.

Observation or excisional biopsy may be appropriate for breast fibroepithelial tumors depending on tumor size, patient age, and clinical features. Carcinoma arising within fibroepithelial tumors has been documented (2), but malignant transformation within bilateral benign breast tumors is exceptionally rare. Clinicians should therefore remain aware that occult carcinoma can occasionally be found within an otherwise benign breast mass, particularly in middle-aged and older women, because this possibility may influence surgical assessment and follow-up. Here, we report a 53-year-old Chinese woman with carcinomas arising within bilateral breast fibroepithelial tumors.

## Case presentation

A 53-year-old Chinese woman presented after incidentally discovering a right breast mass 2 months earlier. She had no breast-related symptoms and initially did not seek medical attention. She was otherwise healthy, with no relevant medical or surgical history. Menarche occurred at 14 years of age, her last menstrual period was in October 2025, and she was perimenopausal at presentation. She was married, had one child, and had breastfed. She reported an allergy to penicillin antibiotics, no history of smoking or alcohol consumption, and no history of anxiety, depression, or other psychiatric disorders. There was no known family history of breast cancer or other gynecologic malignancies.

Physical examination showed symmetric breasts without skin erythema, edema, peau d’orange, or ulceration. Neither nipple was inverted, deviated, or eroded. A firm, mobile mass measuring approximately 10.0 × 8.0 cm was palpable in the upper outer quadrant of the right breast. No definite mass was palpable in the left breast, and no enlarged axillary or supraclavicular lymph nodes were detected bilaterally.

After admission, the patient underwent breast ultrasonography and mammography. Ultrasonography showed a poorly demarcated, irregular, heterogeneous hypoechoic mass measuring 8.6 × 7.8 × 4.2 cm in the 8- to 12-o’clock region of the right breast, with an appearance suggestive of confluent nodules (**Figure 1A**). Multiple hypoechoic nodules were present in the left breast; the largest was located at the 3-o’clock position, 1.0 cm from the nipple, and measured 1.7 × 0.9 cm, with a clear margin and regular shape (**Figure 1B**). No clearly enlarged axillary lymph nodes were identified. The hypoechoic nodules were classified as BI-RADS category 3 on ultrasonography.

**Figure 1:**
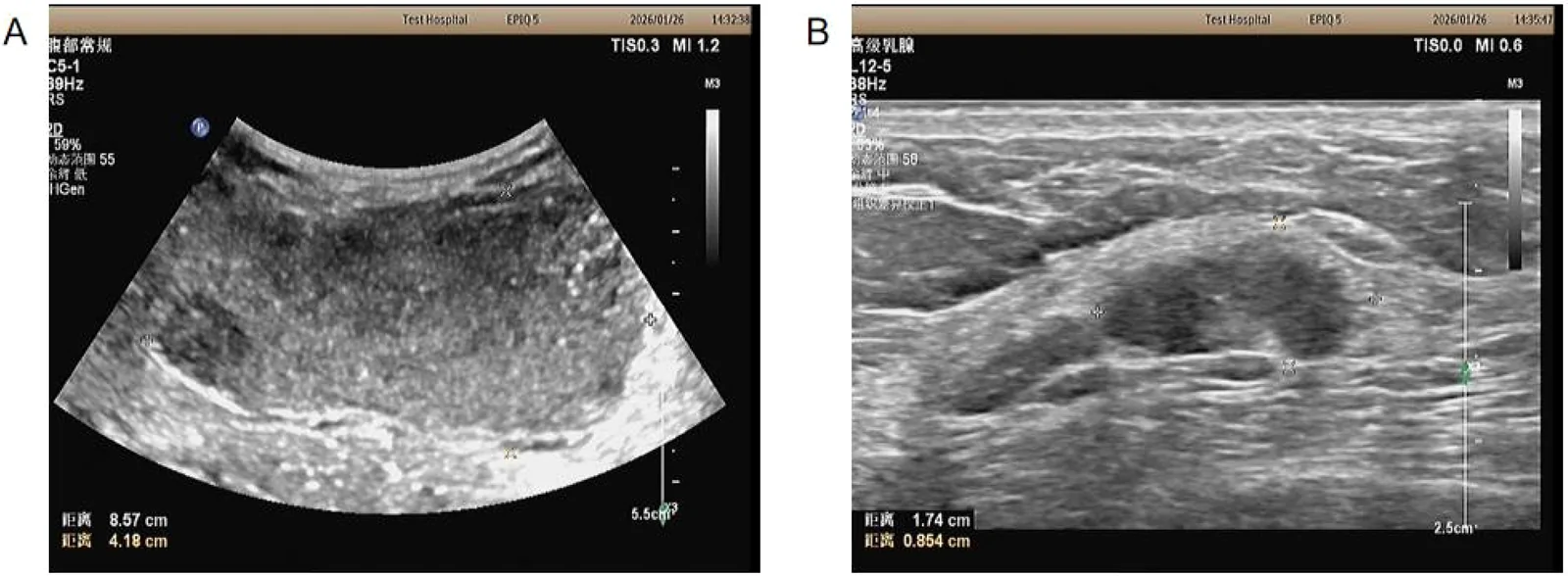
**(A)** Ultrasonography of the right breast showed a heterogeneous hypoechoic lesion measuring 8.57 × 4.18 cm in the 8- to 12-o’clock region, with multiple punctate hyperechoic foci. The final diagnosis was grade 1 invasive carcinoma arising within a benign phyllodes tumor. **(B)** Ultrasonography of the left breast showed a hypoechoic nodule measuring approximately 1.74 × 0.854 cm at the 3-o’clock position, with a mildly irregular shape. The final diagnosis was low-grade DCIS arising within a fibroadenoma.

Mammography demonstrated heterogeneous breast density. A lobulated mass measuring approximately 9.6 × 7.2 cm was present in the upper outer quadrant of the right breast, again suggesting confluent nodules. The margin was clear, and no microcalcifications were observed. Several punctate calcifications were seen in the left breast, and lymph nodes were visible in the anterior axillary regions bilaterally (**Figures 2A–D**). Mammographic assessment was BI-RADS category 4A for the right breast and category 3 for the left breast.

**Figure 2:**
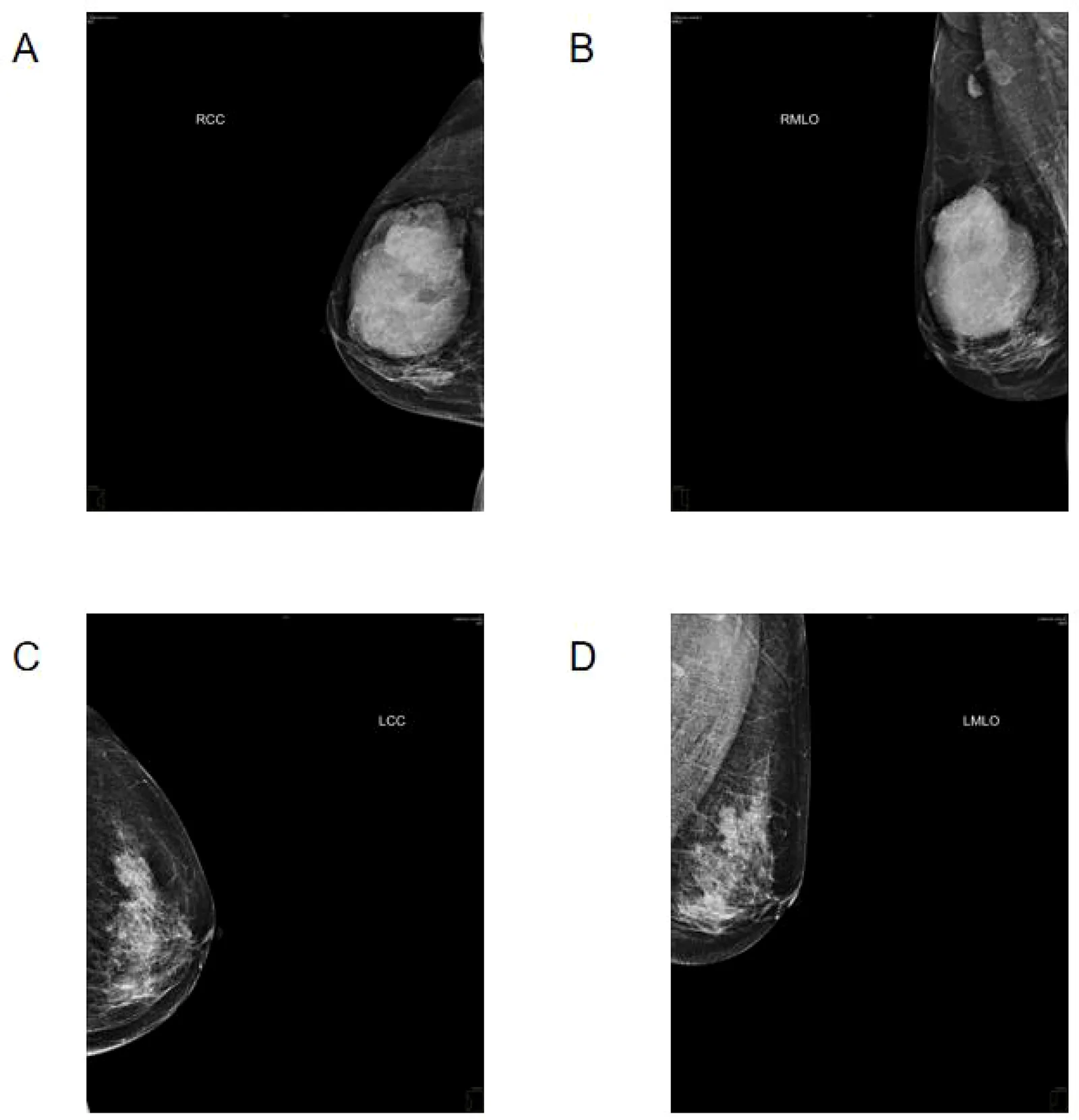
Mammography: **(A)** right craniocaudal view; **(B)** right mediolateral oblique view; **(C)** left craniocaudal view; **(D)** left mediolateral oblique view.

Overall, the dominant right breast mass was considered BI-RADS category 4A, whereas the remaining bilateral lesions were considered BI-RADS category 3.

Based on the physical and imaging findings, breast carcinoma was included in the differential diagnosis. Core needle biopsy was recommended on the day after admission to establish a pathologic diagnosis; however, the patient declined. Surgical excision was therefore selected. After preoperative counseling regarding risks and written informed consent, the patient underwent wide local excision of the right breast mass and ultrasound-guided vacuum-assisted excision of the left breast lesion under general anesthesia the following day.

During excision of the right breast mass, no dense adhesion to the overlying skin or chest wall was noted. The mass was removed intact together with approximately 1 cm of surrounding breast tissue. Superior, inferior, medial, lateral, and deep margin specimens were obtained to confirm the presence of normal tissue around the lesion.

For the left breast lesion, ultrasonography was used to localize the 3-o’clock mass and select an appropriate puncture site. Under real-time ultrasound guidance, the vacuum-assisted biopsy device was advanced behind the lesion, and repeated sector-shaped excisions were performed until the mass was completely removed with a rim of surrounding normal-appearing tissue.

Both breast lesions were submitted for intraoperative frozen-section examination. The enlarged right breast specimen and all surgical margins were also submitted for routine permanent histopathologic examination. Frozen-section findings were interpreted as a benign phyllodes tumor in the right breast and adenosis with fibroadenoma in the left breast, with final evaluation deferred to paraffin sections and immunohistochemistry. The operation was completed uneventfully, and the patient recovered satisfactorily from anesthesia.

### Histopathologic and immunohistochemical findings

Right breast. The mass located at the 8- to 12-o’clock position contained invasive carcinoma of no special type, histologic grade 1, with a maximum invasive tumor diameter of approximately 7 mm. The pathologic stage was pT1Nx. No definite lymphovascular invasion or perineural invasion was identified. The wide local excision specimen and all margins were negative (**Figures 3**–**5**).

**Figure 3:**
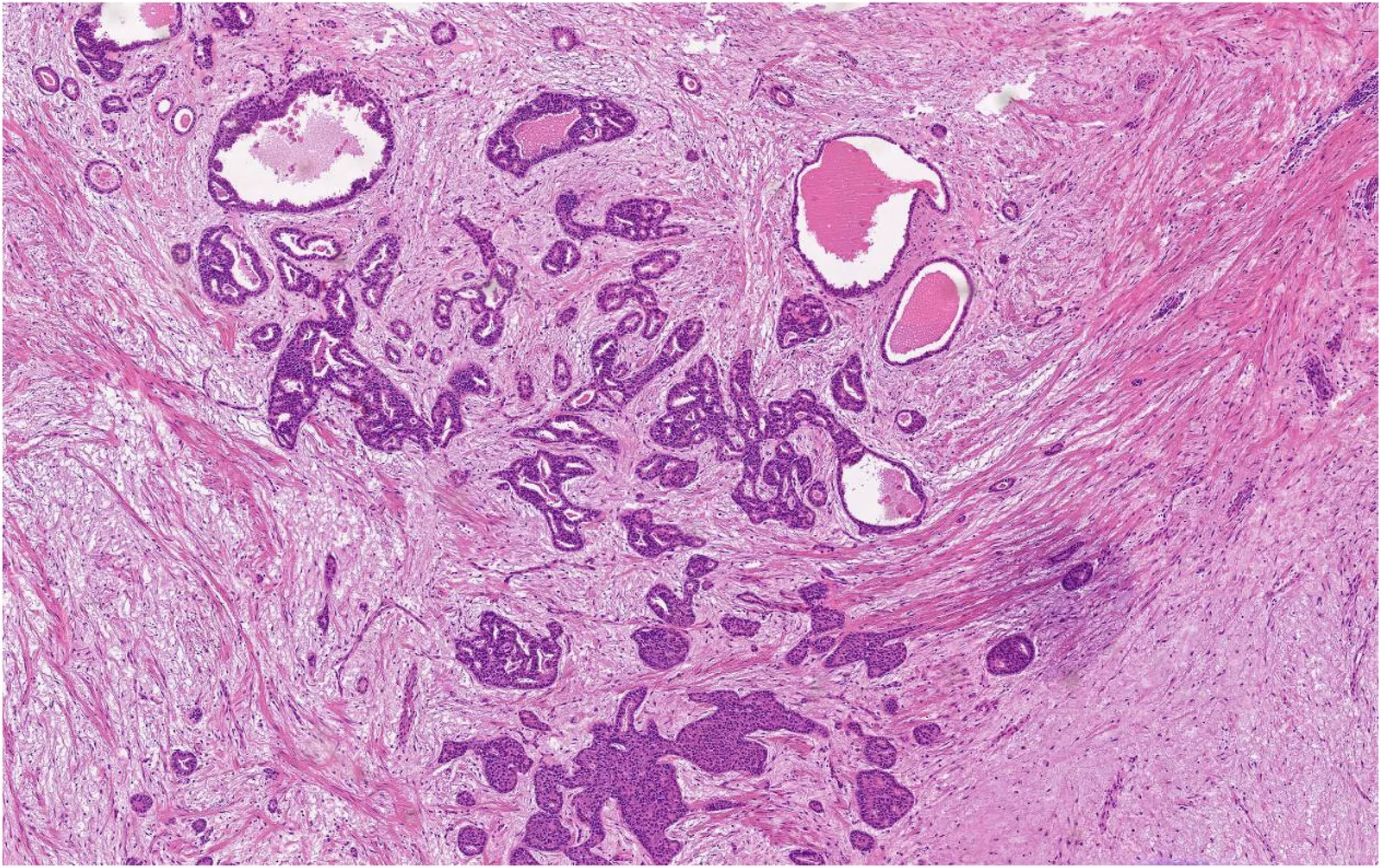
Low-power microscopic view (10×) showing the invasive carcinoma focus within a background benign phyllodes tumor.

**Figure 4:**
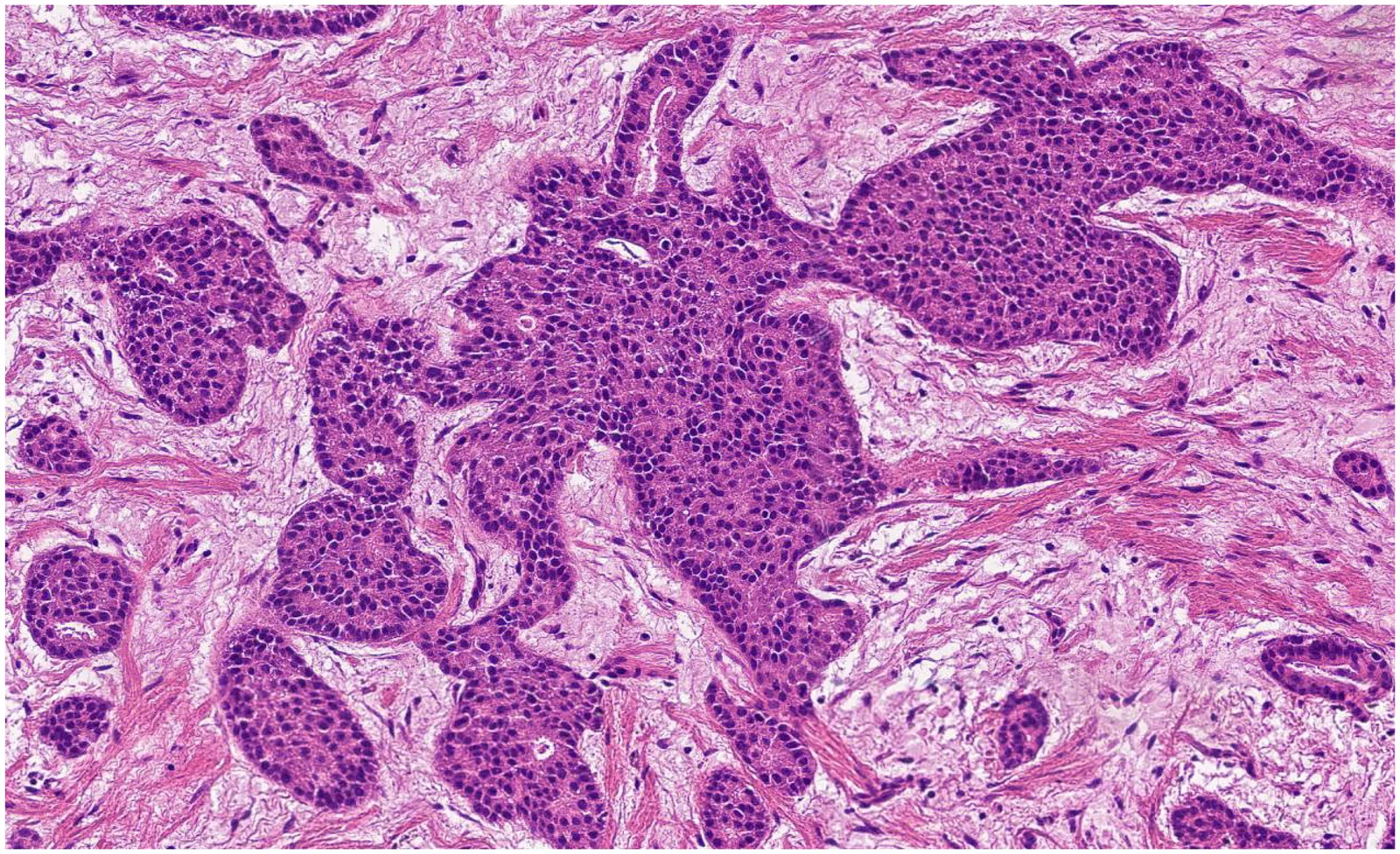
High-power microscopic view (40×) showing invasive carcinoma within the benign phyllodes tumor, with nuclear pleomorphism of the malignant cells.

**Figure 5:**
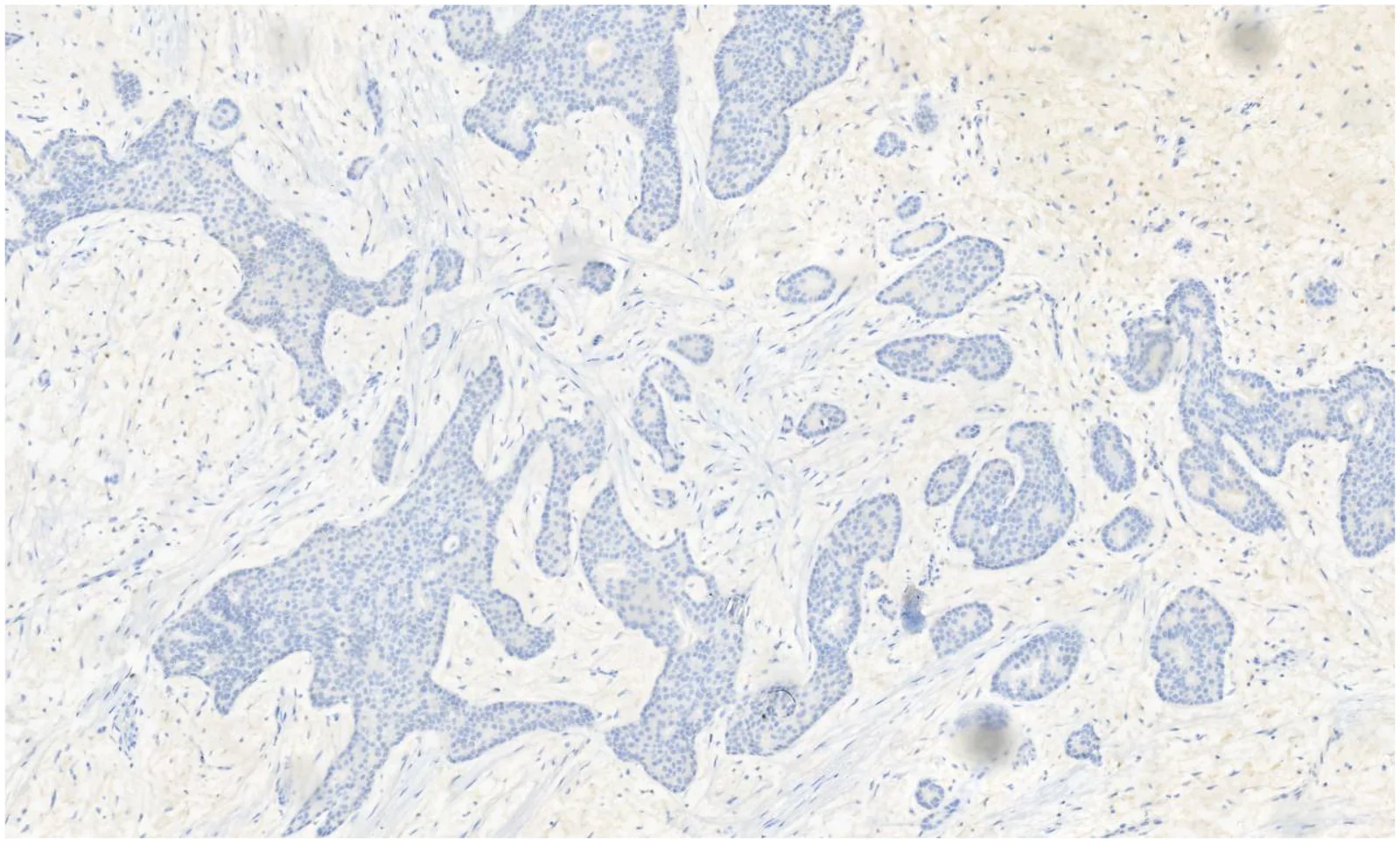
p63 immunohistochemical staining showing absence of myoepithelial staining around the invasive carcinoma.

- Estrogen receptor (ER): 3+, positive.
- Progesterone receptor (PR): 2+ to 3+, positive.
- Human epidermal growth factor receptor 2 (HER2): 1+, low expression.
- Ki-67 proliferation index: approximately 5%.

Left breast. The lesion at the 3-o’clock position contained low-grade DCIS (DCIS), with a maximum diameter of approximately 2.5 mm. The pathologic stage was pTisNx (**Figures 6**–**8**).

- Estrogen receptor (ER): 3+, positive.
- Progesterone receptor (PR): 3+, positive.
- Human epidermal growth factor receptor 2 (HER2): 0, negative.
- Ki-67 proliferation index:approximately 5%.

**Figure 6:**
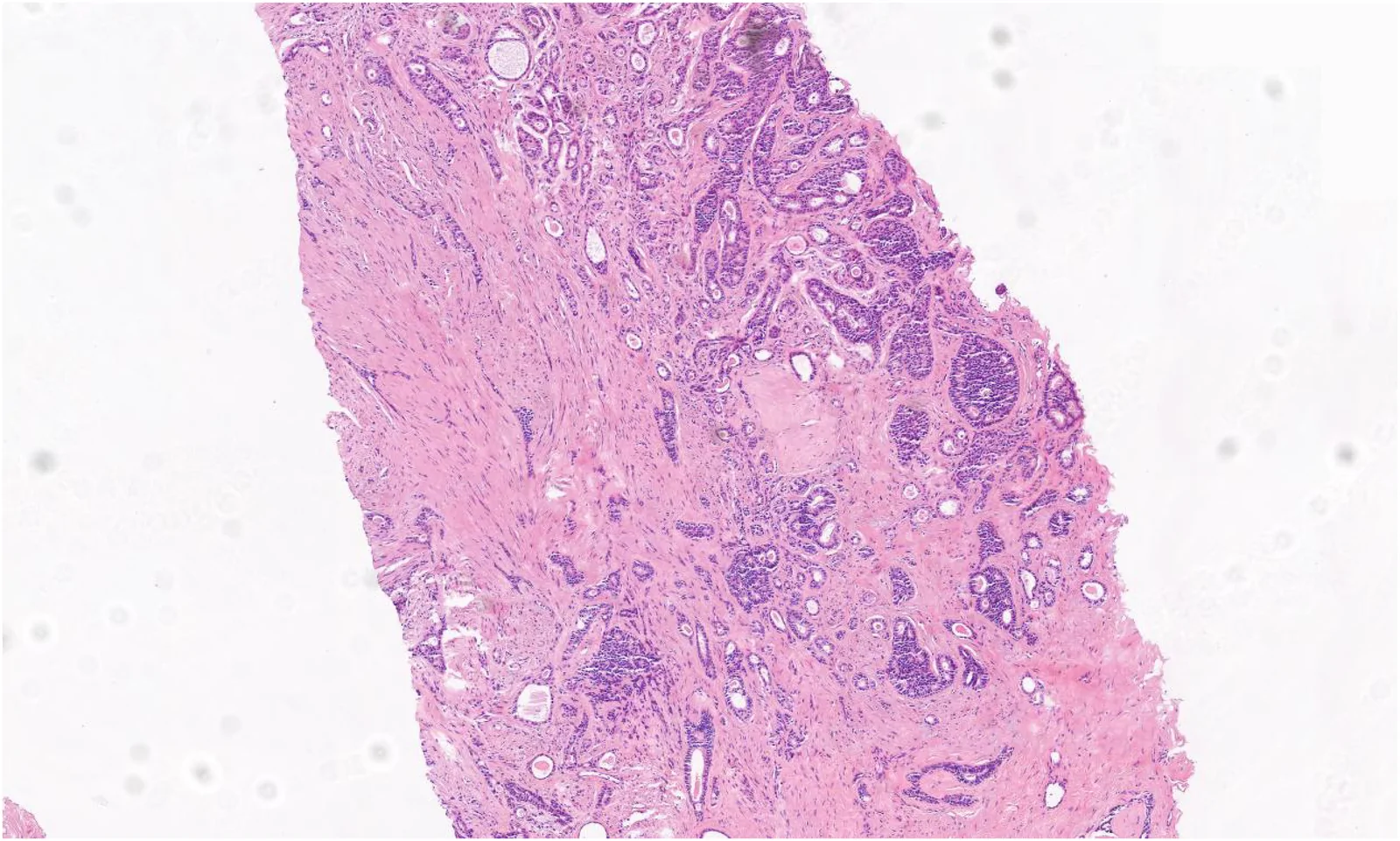
Low-power microscopic view (10×) showing DCIS within a background fibroadenoma.

**Figure 7:**
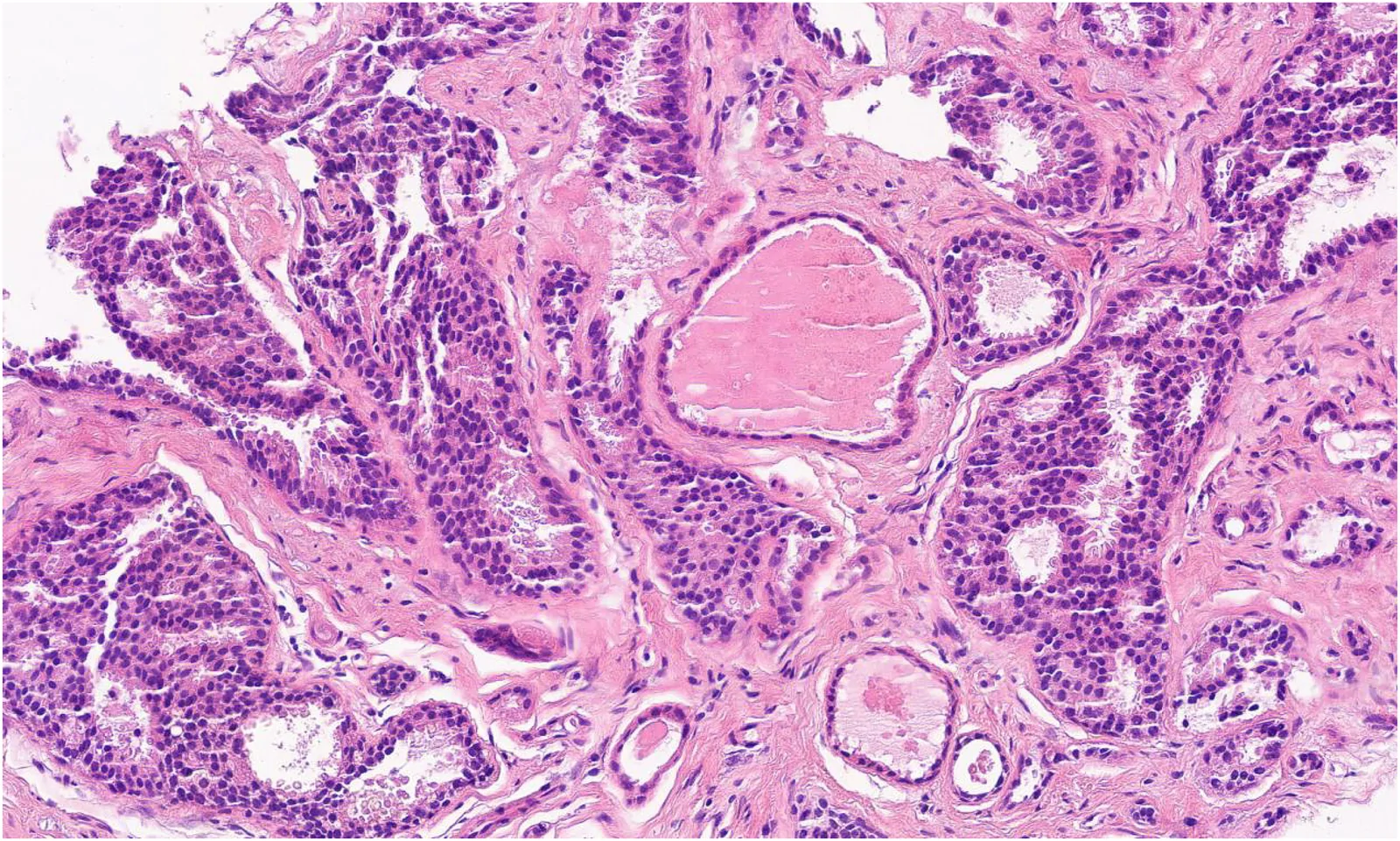
High-power microscopic view (40×) showing a focus of DCIS within the fibroadenoma.

**Figure 8:**
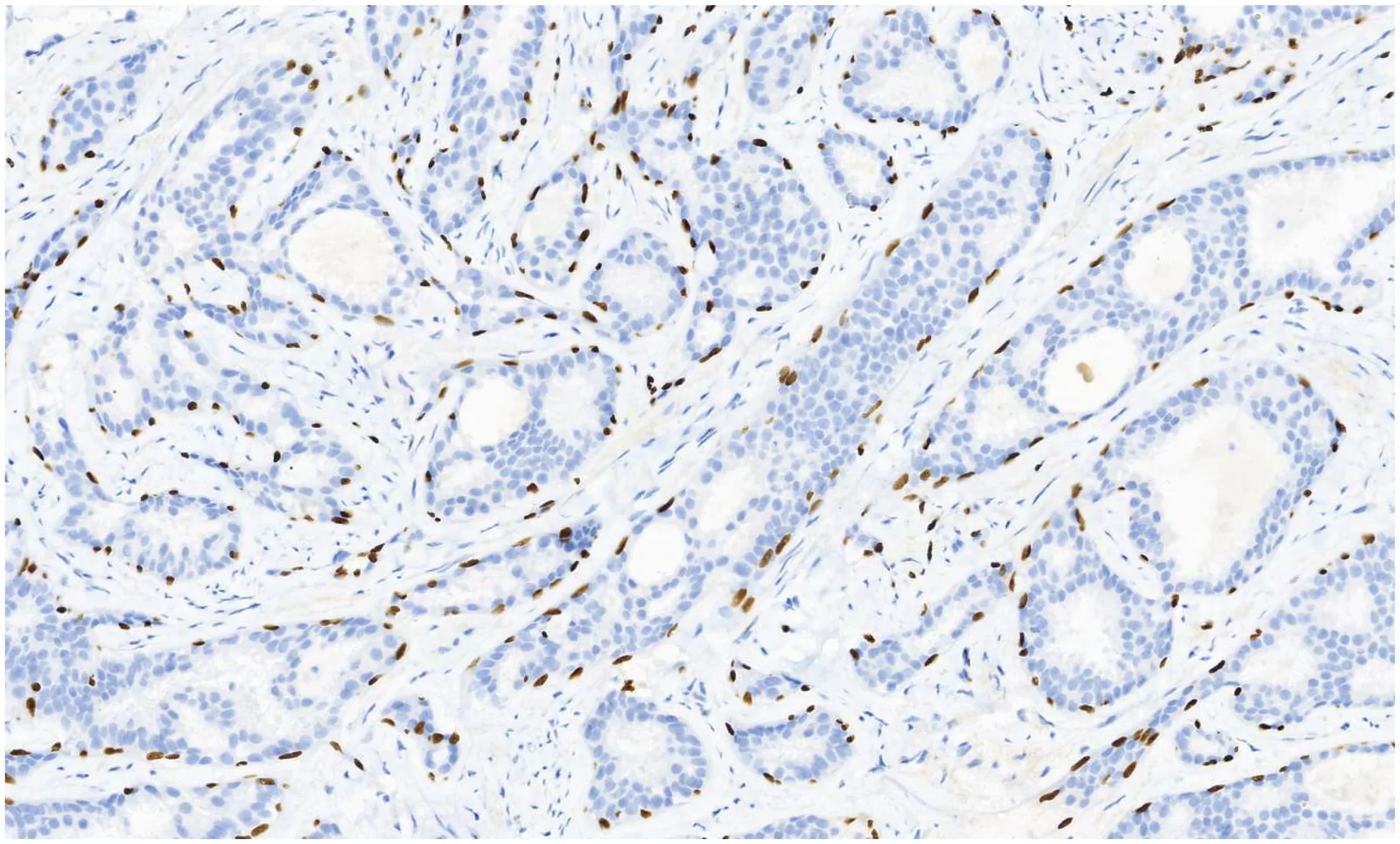
p63 immunohistochemical staining showing preserved myoepithelial staining around the DCIS.

### Multidisciplinary management and follow-up

The case was discussed at a multidisciplinary team(MDT) meeting, with particular attention to whether sentinel lymph node biopsy (SLNB) was required for the right breast lesion and to the subsequent adjuvant treatment strategy. Because the patient had declined preoperative core needle biopsy, the invasive component had not been recognized before surgery and was identified only on postoperative permanent sections with immunohistochemistry.

The MDT considered the biologic risk of the invasive tumor to be very low. Because the overall adjuvant strategy would remain centered on endocrine therapy regardless of axillary nodal status, the potential staging benefit of delayed SLNB was weighed against the risks of an additional operation, including mapping failure, lymphedema, and sensory disturbance. Delayed SLNB of the right axilla was therefore omitted, and close clinical and imaging surveillance was planned.

The left breast lesion consisted of low-grade DCIS without an invasive component and had been completely removed by ultrasound-guided vacuum-assisted excision. Because the risk of axillary nodal metastasis is extremely low in pure DCIS when the lesion has been excised with negative margins, no SLNB was performed for the left axilla. Bilateral axillary surveillance was undertaken clinically and radiologically.

Both malignant foci were ER- and PR-positive. Given the patient’s perimenopausal status, the MDT recommended oral toremifene 60 mg once daily, with planned transition to letrozole 2.5 mg once daily after menopause. The intended total duration of endocrine therapy was 5–10 years, with monitoring of relevant safety parameters, including the endometrium and serum lipid profile.

At 3 months after surgery, breast ultrasonography showed several hypoechoic nodules in both breasts. The largest left-sided nodule was located at the 11-o’clock position and measured approximately 0.8 × 0.7 × 0.4 cm, with a clear margin and mildly irregular shape. The largest right-sided nodule was located at the 7-o’clock position and measured approximately 0.4 × 0.5 × 0.3 cm. No clearly enlarged axillary lymph nodes were observed. All lesions were classified as BI-RADS category 3, and continued toremifene therapy with regular follow-up was recommended **Figure 9**. 

**Figure 9:**
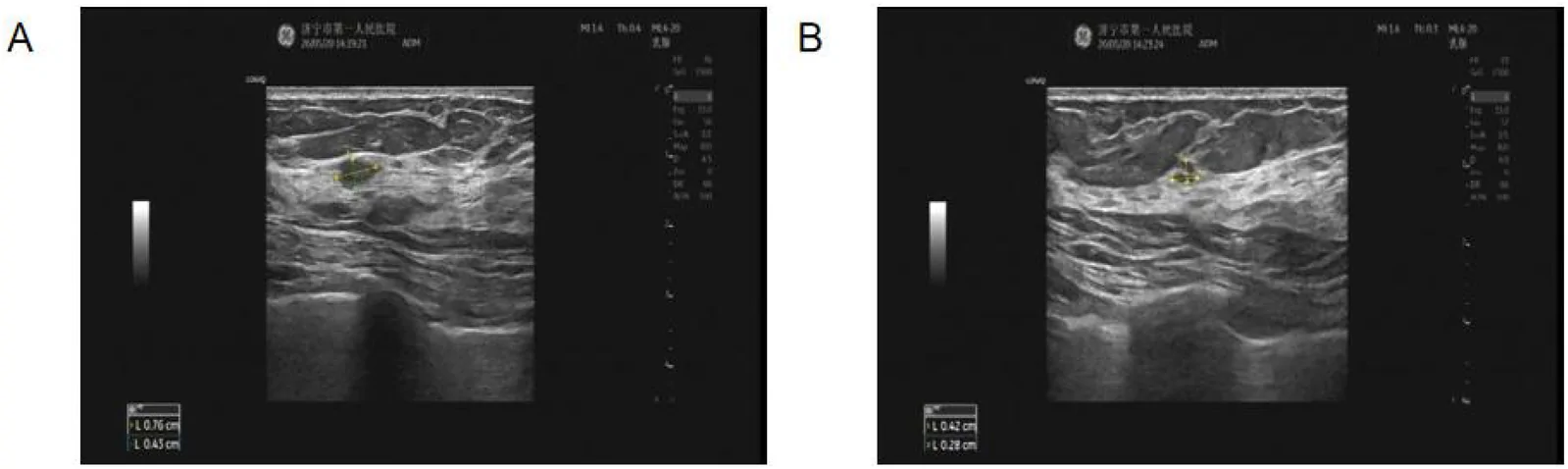
**(A)** The left breast showed multiple hypoechoic nodules with clear margins and mildly irregular shapes; the largest was located at the 11-o’clock position and measured approximately 0.8 × 0.4 cm. **(B)** The right breast showed multiple hypoechoic nodules with clear margins and regular shapes; the largest was located at the 7-o’clock position and measured approximately 0.4 × 0.3 cm.

At 6 months after breast cancer surgery, the patient remained on toremifene, reported regular adherence and good tolerability, and continued scheduled follow-up. No evidence of tumor recurrence had been detected.

## Discussion

Breast fibroepithelial tumors are biphasic neoplasms composed of epithelial and stromal elements. According to the World Health Organization(WHO) classification, they primarily include fibroadenomas and phyllodes tumors. Clinically, they commonly present as painless breast masses, but their treatment and prognosis vary substantially according to the pathologic subtype.

Fibroadenoma is the most common fibroepithelial tumor of the breast and contains both epithelial and mesenchymal components. It generally occurs in younger premenopausal women, although it may become clinically relevant at any age. Ductal epithelial hyperplasia or metaplasia is common within fibroadenomas, whereas malignant transformation is exceptionally uncommon. Carcinoma arising within a fibroadenoma was first described by Cheatle and Cutler in 1931 (3). Most published series report an incidence below 1% (2, 4, 5).

Carcinoma within a fibroadenoma refers to malignant transformation of the epithelial component, with the carcinoma confined to the fibroadenoma or associated with only limited extension beyond it. Reported histologic types include lobular carcinoma *in situ*, ductal carcinoma, and invasive carcinoma (6). Lobular carcinoma *in situ* has been reported most frequently. In the present case, the left breast lesion was confirmed by histopathology and immunohistochemistry to be low-grade DCIS arising within adenosis and fibroadenoma, representing a relatively uncommon pathologic subtype.

The detection rate of DCIS increases with age. Patients with DCIS arising within fibroadenoma have been reported to have a mean age at diagnosis of approximately 40–52 years, roughly two decades older than the age group in which uncomplicated fibroadenoma most commonly occurs (7, 8). Microcalcifications are a typical mammographic feature of DCIS; however, DCIS arising within fibroadenoma may lack characteristic calcifications and may present only as focal architectural distortion or asymmetric density, making diagnosis by mammography alone difficult. Similar cases without mammographically visible microcalcifications have been reported (9).

Breast magnetic resonance imaging (MRI) provides superior soft-tissue contrast, and dynamic contrast-enhanced MRI may aid in distinguishing benign from malignant lesions through enhancement kinetics. Typical fibroadenomas often demonstrate persistent enhancement, whereas a plateau or rapid washout pattern may raise suspicion for malignancy (10). The present patient did not undergo preoperative breast MRI, and the ability of MRI to detect the minute DCIS focus in this case therefore cannot be assessed. Moreover, evidence regarding the reliable detection of very small carcinomas embedded within benign tumors remains limited, and MRI should not be assumed to have identified the occult lesion in this patient.

Phyllodes tumors are rare fibroepithelial tumors, accounting for approximately 0.3%-1% of all breast tumors and occurring most commonly in women aged 40–50 years. They are usually unilateral and may grow rapidly (11). According to WHO criteria and published data, phyllodes tumors are classified as benign, borderline, or malignant according to stromal features, with reported recurrence rates of 10.9%, 14.4%, and 29.6%, respectively; benign phyllodes tumors account for approximately 50%-70% of all phyllodes tumors (12, 13). Invasive ductal carcinoma or invasive carcinoma of no special type arising within a phyllodes tumor is extremely rare, reported in only approximately 1%-2% of cases and more often in association with malignant phyllodes tumors (14). The right breast lesion in the present patient consisted of a benign phyllodes tumor containing a focal grade 1 invasive carcinoma, making this an exceptionally uncommon presentation.

Imaging findings of benign phyllodes tumors and early invasive breast carcinoma can overlap, which complicates preoperative differentiation (14). On mammography, benign phyllodes tumors often appear as well-defined, lobulated masses without malignant calcifications, whereas invasive carcinoma more typically presents as an irregular, ill-defined mass and may be associated with clustered microcalcifications. MRI of phyllodes tumors may demonstrate nonenhancing internal septa and characteristic enhancement patterns related to histologic grade, whereas invasive carcinoma often demonstrates an irregular mass with segmental or regional enhancement and rapid contrast wash-in followed by washout (15). Again, the patient did not undergo preoperative MRI, and the diagnostic value of MRI in this case cannot be determined. Given the small size of the invasive focus (7 mm) and its location within the stroma of a benign phyllodes tumor, an occult lesion could potentially have been missed even if MRI had been performed.

Core needle biopsy is an important preoperative method for determining the nature of a breast mass. However, focal microscopic carcinoma within a fibroepithelial tumor may be missed because the malignant component represents only a small proportion of the lesion and sampling may capture only benign stromal or epithelial tissue. Many reported carcinomas arising within fibroadenomas or phyllodes tumors have therefore been discovered incidentally on permanent sections with immunohistochemistry after excision. In the present case, the patient declined preoperative core needle biopsy and proceeded directly to surgery; the malignant foci were ultimately diagnosed on postoperative pathologic examination, underscoring the sampling limitations relevant to occult, minute malignant components.

Surgery is the principal treatment for breast fibroepithelial tumors. Complete excision is generally sufficient for benign fibroadenoma. For benign phyllodes tumors, wider excision has traditionally been recommended to reduce the risk of local recurrence, and mastectomy may be considered for very large, multicentric, or centrally located lesions (8, 14). In this patient, the right breast lesion was large (approximately 8.6 × 7.8 × 4.2 cm) and appeared multinodular and confluent; wide local excision was therefore performed. The largest left breast lesion measured approximately 1.7 × 0.9 cm and was considered suitable for ultrasound-guided vacuum-assisted excision.

Because carcinoma arising within fibroepithelial tumors is rare, no standardized management pathway specifically addresses all such cases. In clinical practice, treatment is generally individualized according to the stage and molecular subtype of the malignant component, using established breast cancer principles as a reference. In the present patient, the right-sided invasive carcinoma measured 7 mm, was histologic grade 1, hormone receptor positive, HER2-low, had a Ki-67 index of approximately 5%, and showed no lymphovascular invasion. The MDT therefore considered its biologic behavior to be low risk.

Regarding axillary staging, the MDT weighed the expected benefit of delayed SLNB against the morbidity of a second operation. Even if occult axillary micrometastasis had been present, the overall adjuvant treatment strategy was expected to remain endocrine therapy, and nodal staging was not considered likely to alter management. For the left breast, the lesion was pure low-grade DCIS without invasive carcinoma. Current clinical guidance generally does not recommend routine SLNB for breast-conserving treatment of pure DCIS; SLNB is more commonly considered when mastectomy is planned or when invasive disease is subsequently identified (16). Consequently, no SLNB was performed on either side, and both axillae were followed clinically and radiologically.

Adjuvant endocrine therapy is indicated for hormone receptor-positive breast malignancies. Selective estrogen receptor modulators may be used in premenopausal or perimenopausal women, with aromatase inhibitors becoming appropriate after menopause (17). Because both malignant foci in this patient were ER- and PR-positive, toremifene was selected during the perimenopausal period, with planned transition to letrozole after menopause and a total treatment duration of 5–10 years. This strategy was intended to address both the right-sided invasive carcinoma and the left-sided DCIS.

Malignant transformation within bilateral benign breast fibroepithelial tumors is exceedingly rare. Preoperative imaging may lack typical malignant features, and needle biopsy may miss small malignant foci. Definitive diagnosis may require careful examination of the complete excision specimen with permanent histopathology and immunohistochemistry. Because high-level evidence is lacking, clinical management should be individualized through multidisciplinary assessment, including consideration of tumor characteristics, patient factors, axillary staging, systemic therapy, and the balance of procedural benefits and risks. This report is limited by its single-patient design and short follow-up period.

## Conclusion

Carcinoma arising within bilateral benign breast fibroepithelial tumors is exceptionally rare. In middle-aged and older patients, imaging may show no typical malignant features and needle sampling may fail to detect small occult malignant foci; definitive diagnosis may therefore depend on postoperative histopathologic examination. Surgical excision remains the foundation of treatment, while adjuvant therapy should be individualized according to pathologic subtype and molecular phenotype, followed by long-term surveillance. Because this report describes a single patient with limited follow-up, the management approach should not be generalized as a universal treatment recommendation.

## Data Availability

The datasets presented in this article are not readily available because the patient has been discharged from the hospital and could not be contacted in a timely manner. Requests to access the datasets should be directed to the dataset contains anonymized clinical patient information. Data sharing is restricted due to patient−privacy protection and institutional data−management regulations of Jining No 1 People’s Hospital. Raw clinical data cannot be publicly released.
